# Deferasirox, a novel oral iron chelator, shows antiproliferative activity against pancreatic cancer in vitro and in vivo

**DOI:** 10.1186/s12885-016-2744-9

**Published:** 2016-08-31

**Authors:** Hirofumi Harima, Seiji Kaino, Taro Takami, Shuhei Shinoda, Toshihiko Matsumoto, Koichi Fujisawa, Naoki Yamamoto, Takahiro Yamasaki, Isao Sakaida

**Affiliations:** 1 Department of Gastroenterology and Hepatology, Yamaguchi University Graduate School of Medicine, 1-1-1 Minami-Kogushi, Ube, Yamaguchi 755-8505 Japan; 2 Department of Oncology and Laboratory Medicine, Yamaguchi University Graduate School of Medicine, 1-1-1 Minami-Kogushi, Ube, Yamaguchi 755-8505 Japan

**Keywords:** Deferasirox, Iron chelator, Pancreatic cancer

## Abstract

**Background:**

Iron is essential for cell replication, metabolism and growth. Because neoplastic cells have high iron requirements due to their rapid proliferation, iron depletion may be a novel therapeutic strategy for cancer. Deferasirox (DFX), a novel oral iron chelator, has been successful in clinical trials in iron-overload patients and has been expected to become an anticancer agent. However, no studies have investigated the effects of DFX on pancreatic cancer. This study aimed to elucidate the effects of DFX against pancreatic cancer.

**Methods:**

The effects of DFX on cell cycle, proliferation, and apoptosis were examined in three human pancreatic cancer cell lines: BxPC-3, HPAF-II, and Panc 10.05. The effect of orally administered DFX on the growth of BxPC-3 pancreatic cancer xenografts was also examined in nude mice. Additionally, microarray analysis was performed using tumors excised from xenografts.

**Results:**

DFX inhibited pancreatic cancer cell proliferation in a dose-dependent manner. A concentration of 10 μM DFX arrested the cell cycle in S phase, whereas 50 and 100 μM DFX induced apoptosis. In nude mice, orally administered DFX at 160 and 200 mg/kg suppressed xenograft tumor growth with no serious side effects (*n* = 5; average tumor volumes of 674 mm^3^ for controls vs. 327 mm^3^ for 160 mg/kg DFX, *p* <0.05; average tumor volumes of 674 mm^3^ for controls vs. 274 mm^3^ for 200 mg/kg DFX, *p* <0.05). Importantly, serum biochemistry analysis indicated that serum levels of ferritin were significantly decreased by the oral administration of 160 or 200 mg/kg DFX (*n* = 5; average serum ferritin of 18 ng/ml for controls vs. 9 ng/ml for 160 mg/kg DFX, *p* <0.05; average serum ferritin of 18 ng/ml for controls vs. 10 ng/ml for 200 mg/kg DFX, *p* <0.05). Gene expression analysis revealed that most genes in pancreatic adenocarcinoma signaling, especially transforming growth factor-ß1 (TGF-ß1), were downregulated by DFX.

**Conclusions:**

DFX has potential as a therapeutic agent for pancreatic cancer. Iron depletion was essential for the antiproliferative effect of DFX in a preclinical model, and DFX acted through the suppression of TGF-ß signaling.

## Background

Pancreatic cancer is the fifth leading cause of cancer-related deaths, and the number of cases has been increasing in Japan [1]. It is the fifth and fourth leading cause of cancer-related deaths in Europe and in North America, respectively [2]. Pancreatic cancer is associated with the worst prognosis among solid tumors [3]; the 5-year survival rate of pancreatic cancer, including resectable cases, is not more than 10 % [4]. Surgical resection is the only potential curative therapy, but many patients with pancreatic cancer are not candidates for surgical resection at the time of diagnosis. For patients with unresectable pancreatic cancer, chemotherapy is recommended as the current standard care [5]. During the last two decades, gemcitabine has been the standard chemotherapy for pancreatic cancer. Recently, new combination chemotherapies have been developed, such as regimens combining fluorouracil, irinotecan, oxaliplatin, and leucovorin (FOLFIRINOX) or albumin-bound paclitaxel with gemcitabine [6, 7]. However, while combination chemotherapies have shown therapeutic advantages over single-agent gemcitabine, they also have a high incidence of side effects. In addition, more than half of pancreatic cancer patients are diagnosed at an age of 65 years or older [4]. Therefore, a new chemotherapeutic strategy for pancreatic cancer is required for these patients with refractory chemotherapy due to side effects and/or advanced age.

Iron is essential for cell replication, metabolism and growth [8]. Because neoplastic cells have high iron requirements due to their rapid proliferation, iron depletion could be a novel therapeutic strategy for cancer [9]. Although iron chelators, which are commonly used for treating iron-overload disease, are not classified as anticancer drugs; they exert antiproliferative effects in several cancers [10–12]. We have reported that deferoxamine (DFO), a standard iron chelator, can prevent the development of liver preneoplastic lesions in rats [13]. We also performed a pilot study using DFO in advanced hepatocellular carcinoma patients and reported the efficacy of this iron chelator [14]. Considering the mechanism of action of iron chelators as anticancer agents, as well as other cancers, iron chelators are thought to be effective pancreatic cancer treatments. Kovacevic et al. reported that thiosemicarbazone iron chelators inhibited pancreatic cancer growth in vitro and in vivo [15]. Therefore, iron chelators represent a potential therapeutic strategy for pancreatic cancer. However, most iron chelators, including DFO and thiosemicarbazones, cannot be administered orally, thus limiting their clinical application.

Recently, deferasirox (DFX), a newly developed oral iron chelator, was successful in clinical trials in iron-overload disease patients and has been implemented as an alternative to DFO [16]. A number of in vitro and in vivo studies have demonstrated that DFX has powerful antiproliferative effects [17]. To our knowledge, there have been no studies investigating the effects of DFX against pancreatic cancer. Therefore, this study aimed to evaluate the antiproliferative activity of DFX against pancreatic cancer in vitro and in vivo.

## Methods

### Cell culture

The pancreatic cancer cell lines BxPC-3, HPAF-II, and Panc 10.05 were obtained from the American Type Culture Collection (Manassas, VA, USA). BxPC-3 and Panc 10.05 cells are epithelial cell lines that were derived from pancreatic adenocarcinomas. The HPAF-II cell line consists of epithelial cells derived from ascites that originated from pancreatic adenocarcinomas.

BxPC-3 cells were grown in RPMI-1640 (Life Technologies, Carlsbad, CA, USA) with 10 % (v/v) fetal calf serum. HPAF-II cells were grown in Eagle’s medium (Life Technologies) with 10 % (v/v) fetal calf serum. Panc 10.05 cells were grown in RPMI-1640 (Life Technologies) containing 10 units/ml of human recombinant insulin, and 15 % (v/v) fetal calf serum. All media were supplemented with 50 μg/ml gentamicin. All cells were incubated at 37 °C in a humidified atmosphere containing 5 % CO_2_.

### Reagents

The oral iron chelator DFX was obtained from Novartis (Basel, Switzerland). For in vitro studies, DFX was dissolved in dimethyl sulfoxide at a stock concentration of 100 mM and was used at the concentrations indicated in the results and figures by dilution in culture media containing 10 % fetal calf serum. For in vivo studies, DFX was dissolved in sodium chloride solution (0.9 % w/v; Chemix Inc., Shinyokohama Kohoku-ku, Yokohama, Japan).

### Cell proliferation

Cellular proliferation was examined using the 3-(4,5-dimethylthiazol-2-yl)-5-(3-carboxymethoxyphenyl)-2-(4-sulfophenyl)-2H-tetrazolium, inner salt (MTS) assay. Cell suspensions (2,000 cells/100 μl) were added to each well in a 96-multiwell culture plate (BD Bioscience, San Jose, CA, USA) and incubated at 37 °C for 24 h. The indicated concentrations of DFX were then added to each well, and the cells were incubated for a further 72 h. At the end of the culture period, 10 μl of MTS solution (Promega, Madison, WI, USA) was added to each 100 μl of culture media and incubated for 2 h. Absorbance at 490 nm was measured with a multimode reader (Infinite 200 PRO, Tecan Trading, AG, Switzerland), and the results are expressed as the percentage viable with respect to the untreated control.

### Cell cycle analysis

Each pancreatic cancer cell line was seeded into 100-mm dishes and cultured with phosphate-buffered saline (PBS) as a vehicle control or DFX at 10, 50, or 100 μM for 72 h. After incubation, the cells were fixed with 70 % ethanol and stored overnight at −20 °C. The cells were washed and then stained with a solution containing 0.1 % Triton® X-100 (Promega), 0.02 mg/ml propidium iodide (PI; Sigma-Aldrich, St. Louis, MO, USA), and 0.2 mg/ml RNase A (Qiagen, Hilden, Germany) in the dark at 37 °C for 15 min. After staining, the cells were subjected to cellular DNA content examination by a flow cytometer (Gallios, Beckman Coulter, Fullerton, CA, USA). The data were analyzed by Multicycle for Windows software (Beckman Coulter).

### Apoptosis analysis by flow cytometry

For the apoptosis analysis, the cells were cultured as described above. After harvesting, apoptosis was evaluated with an apoptosis detection kit (Annexin V Apoptosis Detection Kit APC, eBioscience, San Diego, CA, USA) according to the manufacturer’s instructions. After staining, the cells were examined using a flow cytometer (Gallios, Beckman Coulter). The data were analyzed by FlowJo software (Tree star, Ashland, OR, USA).

### Apoptosis analysis with the luminescence assay

Cell suspensions (2,000 cells/100 μl) were added to each well of a 96-multiwell culture plate (BD Bioscience) and were incubated at 37 °C for 24 h. PBS as a vehicle control or the indicated concentrations of DFX were then added to each well, and the cells were further incubated for 48 h. Immediately after the incubation, caspase activity was measured using the caspase 3/7 assay kit (Caspase-Glo 3/7 kit, Promega) according to the manufacturer’s instructions.

### Tumor xenografts in nude mice and deferasirox administration

Animal care was performed in accordance with the animal ethics requirements at Yamaguchi University School of Medicine, and the experimental protocol was approved (approval ID 21-035). Twenty female BALB/c (nu/nu) mice were purchased from Nippon SLC (Shizuoka, Japan) and were housed in sterile conditions. Experiments commenced when the mice were 8–10 weeks of age. Tumor cells (BxPC-3) in culture were harvested and resuspended in a 1:1 ratio of RPMI-1640 and Matrigel (BD Bioscience). Viable cells (5 × 10^6^ cells) were injected subcutaneously into the backs of the mice. After engraftment, tumor size was measured using Vernier calipers every 2 days, and tumor volume was calculated as follows: tumor volume (mm^3^) = (the longest diameter) (mm) × (the shortest diameter) (mm)/2. When tumor volumes reached 150 mm^3^, oral treatment began (day 0). Each group of mice (*n* = 5) received DFX suspended in saline, which was administered by oral gavage every second day, with three treatments per week, over 21 days at concentrations of 120, 160, or 200 mg/kg. The control mice were treated with the vehicle alone. At the end of the experiment, the mice were sacrificed, and the tumors were excised and processed for immunohistochemistry and genetic analyses. A total of 20 blood samples were collected simultaneously during tumor removal. Serum levels of ferritin were measured using the enzyme-linked immunoassay method (Mouse Ferritin ELISA kit, Kamiya Biochemical Company, Seattle, WA, USA). Serum biochemistry with the exception of ferritin was analyzed by YAMAGUCHI Laboratory Co., Ltd. (Ube, Japan).

### Immunohistochemistry

The removed tumors were fixed in 4 % paraformaldehyde (Muto-kagaku, Tokyo, Japan), sectioned, and embedded in paraffin. Immunohistochemistry was performed as previously described on the paraffin sections with antibody specific to ferritin-H (Anti-Ferritin Heavy Chain antibody, AbCam, Cambridge, MA, USA) [18]. The slides were scored according to the intensity of the immunoreactivity and the percentage of epithelial cells stained [19].

### The detection of gene expression alternation in resected tumors induced by deferasirox administration

#### Total RNA isolation

A total of six tumors were genetically analyzed. Of these, three tumors were removed from vehicle-treated mice, and the other three tumors were removed from DFX 200 mg/kg-treated mice. According to the manufacturer’s instructions, total RNA was isolated from the removal tumors using TRIzol Reagent (Invitrogen Corp., CA, USA) and purified using the SV Total RNA Isolation System (Promega). RNA samples were quantified using a NanoDrop ND-1000 spectrophotometer (Thermo Fisher Scientific Inc., Wilmington, DE, USA), and RNA quality was checked using an Experion automated electrophoresis station (Bio-Rad Laboratories Inc., Hercules, CA, USA).

### Gene expression microarrays

The cRNA was amplified, labeled, and hybridized to a 60K Agilent 60-mer oligomicroarray according to the manufacturer’s instructions. All hybridized microarray slides were scanned by an Agilent scanner. Relative hybridization intensities and background hybridization values were calculated using Agilent Feature Extraction Software (9.5.1.1).

### Data analysis and filter criteria

The raw signal intensities of all samples were log_2_-transformed and normalized with a quantile algorithm from the ‘preprocessCore’ library package [20] on Bioconductor software [21]. We selected the probes, excluding the control probes, where the detection *p*-values of all samples were less than 0.05, and used them to identify differentially expressed genes. To determine significant enrichment canonical pathways, we used the tools and data provide by the Ingenuity Pathway Analysis (IPA) (Ingenuity Systems, INC. http://www.ingenuity.com). The results are the comparisons of tumors removed from vehicle-treated mice vs. the tumors removed from DFX 200 mg/kg-treated mice.

### Statistical analyses

All obtained data are calculated and expressed as the mean ± SD. In the in vitro experiments, the differences were analyzed statistically using 1-way ANOVA, followed by Dannett’s test. In the in vivo experiments, the differences were analyzed statistically using the Kruskal-Wallis H test, followed by Steel’s test. JMP 9 statistical software (SAS Institute Inc., Cary, NC, USA) was used in the analysis. Values of *p* <0.05 were considered significant.

## Results

### DFX inhibited cell proliferation in pancreatic cancer cell lines

**Fig. 1:**
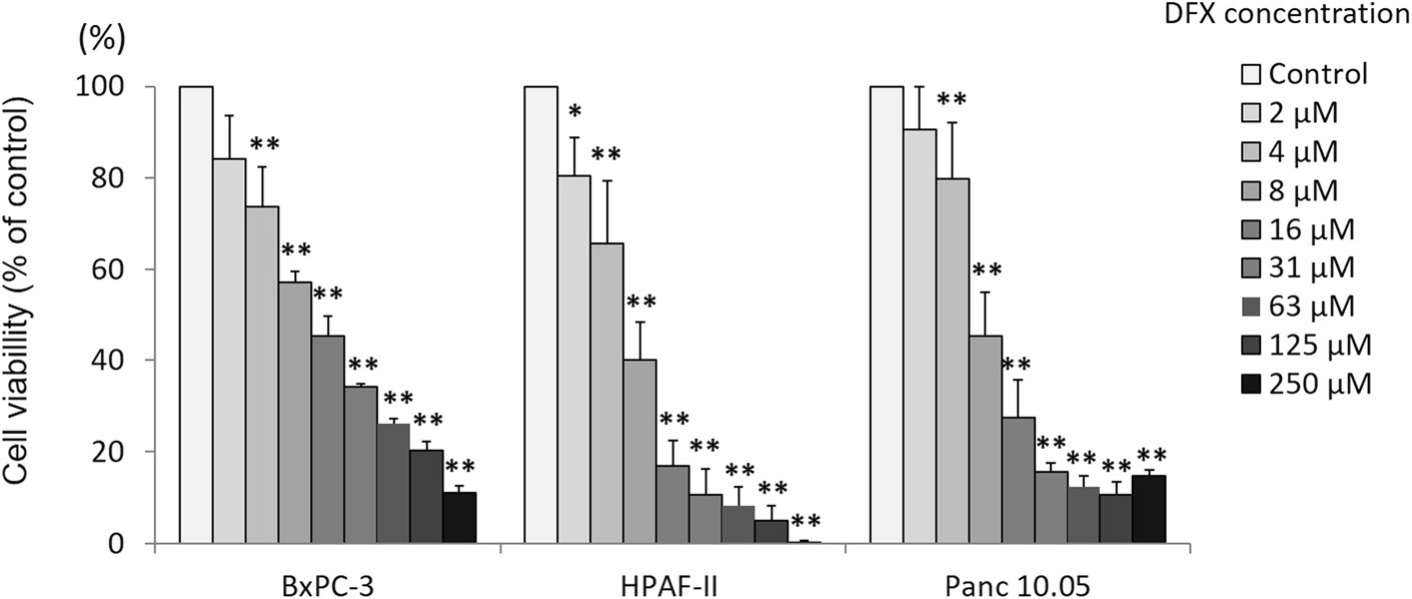
DFX inhibited the proliferation of pancreatic cancer cell lines. Cell proliferation was measured using the MTS assay after cells were treated with DFX 72 h. The viability of BxPC-3, HPAF-II, and Panc 10.05 cells incubated with DFX decreased in a dose-dependent manner. The data are presented as the mean ± SD (*n* = 3–5). *p <0.05, **p <0.01 vs. control

To examine the antiproliferative activity of DFX against pancreatic cancer in vitro, the pancreatic cancer cell lines BxPC-3, HPAF-II, and Panc 10.05 were incubated with either vehicle control (PBS) or the indicated concentrations of DFX for 72 h; then, the cell survival rates were measured using the MTS assay. The cell survival rates are shown in Fig. 1. Incubation of all three cell lines with DFX inhibited cellular proliferation in a dose-dependent manner. DFX had the same level of antiproliferative activity in all three cell lines. As indicated in Table 1, the IC_50_ values for the BxPC-3, HPAF-II, and Panc 10.05 pancreatic cancer cell lines were 7.3 ± 1.0, 5.6 ± 1.0, and 6.1 ± 0.2 μM, respectively. There were no significant differences in the IC_50_ values of each pancreatic cancer cell line.

**Table 1:** IC_50_ values of DFX in three pancreatic cancer cell lines after a 72-h incubation

	BxPC-3	HPAF-II	Panc 10.05
IC_50_ (μM)	7.3 ± 1.0	5.6 ± 1.0	6.1 ± 0.2

### DFX arrested the cell cycle at the S phase in pancreatic cancer cell lines

**Fig. 2:**
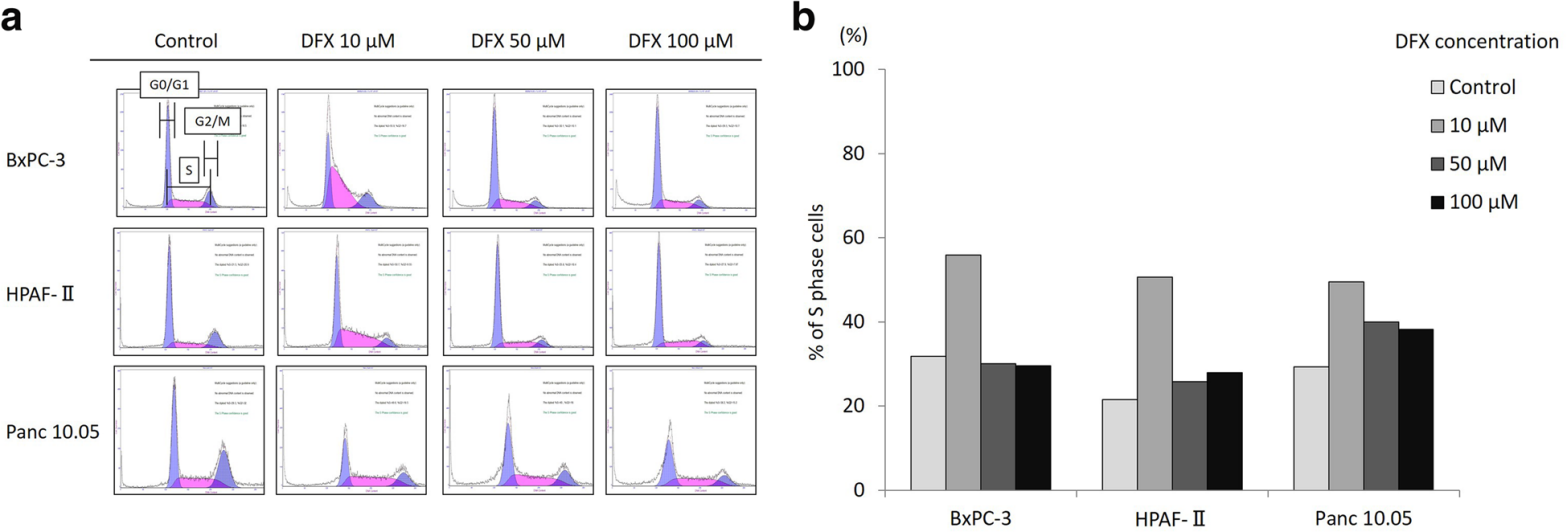
DFX arrested the cell cycle at the S phase in pancreatic cancer cell lines. **a** BxPC-3, HPAF-II, and Panc 10.05 cells were incubated with the vehicle control (PBS) or DFX at concentrations of 10, 50, or 100 μM for 72 h. The cell cycle phase of the treated cells was examined by flow cytometry. The percentages of S phase cells are highlighted in pink. **b** The percentages of S phase cells in each concentration of DFX are shown. When the cells were treated with 10 μM DFX, the number of cells in S phase increased in all three cell lines (*n* = 1)

To explore the mechanism of the antiproliferative activity of DFX, the pancreatic cancer cell lines BxPC-3, HPAF-II, and Panc 10.0 were incubated with either the vehicle control (PBS) or 10, 50, or 100 μM concentrations of DFX for 72 h, and the cell cycle was examined with flow cytometry using PI staining. The analyzed results are shown in Fig. 2a, and the percentage of S phase cells are highlighted in pink. The percentage of S phase cells for each concentration of DFX is shown in Fig. 2b. In all three cell lines, the percentage of S phase cells incubated with 10 μM concentration of DFX was increased. These results demonstrated that 10 μM DFX arrested the cell cycle of pancreatic cancer cells in S phase.

### DFX induced apoptosis in pancreatic cancer cell lines

**Fig. 3:**
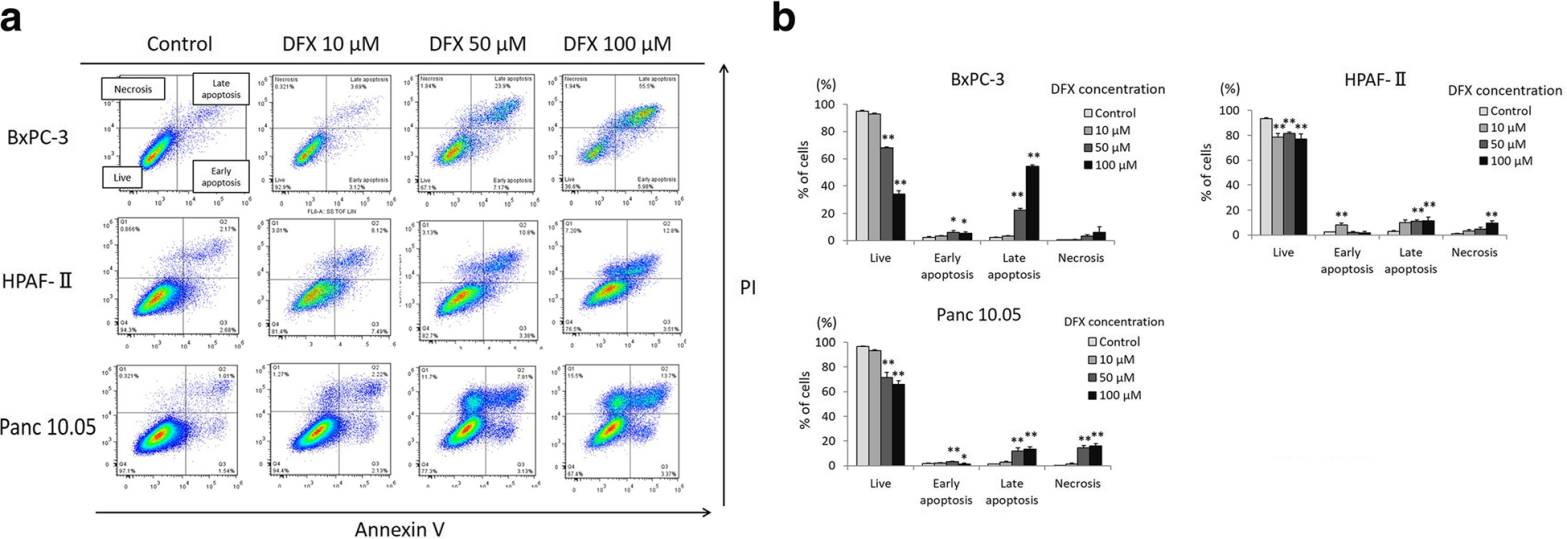
DFX induced apoptosis in pancreatic cancer cell lines. **a** BxPC-3, HPAF-II, and Panc 10.05 cells were incubated with the vehicle control (PBS) or DFX at 10, 50, or 100 μM for 72 h. DFX-treated BxPC-3, HPAF-II, and Panc 10.05 cells were stained with Annexin V/PI and examined by flow cytometry. **b** The percentages of live, apoptotic, and necrotic cells are presented as the mean ± SD (*n* = 3). *p <0.05, **p <0.01 vs. control

**Fig. 4:**
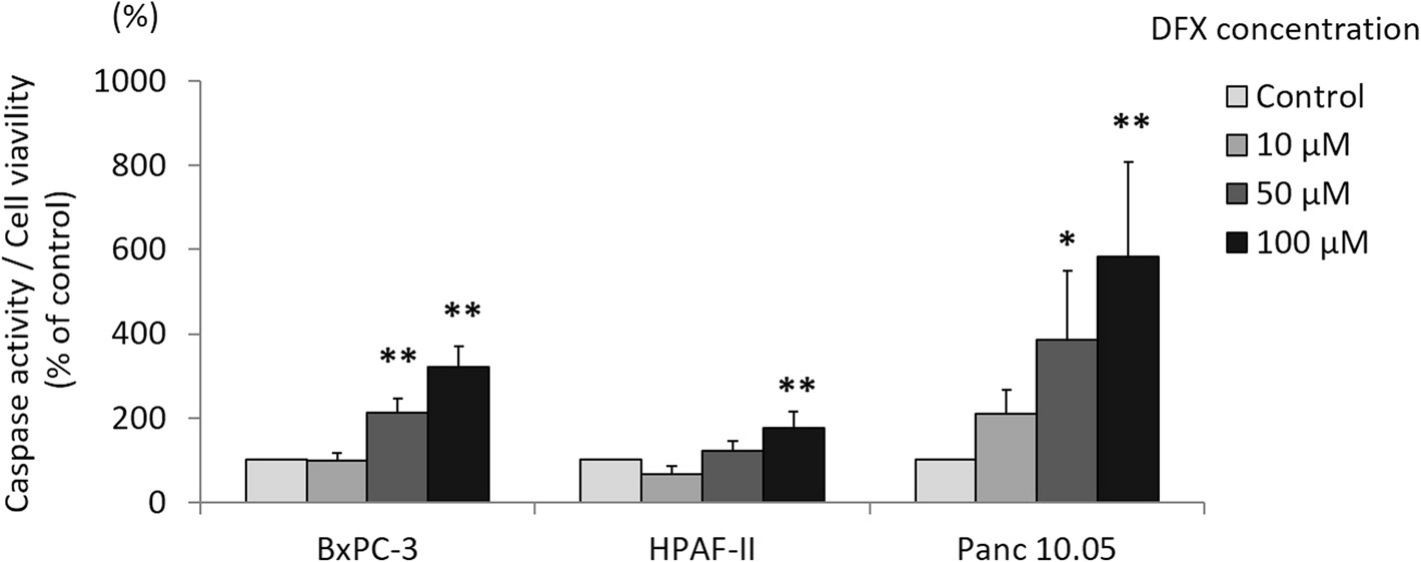
DFX increased caspase 3/7 activity in pancreatic cancer cell lines. BxPC-3, HPAF-II, and Panc 10.05 cells were incubated with the vehicle control (PBS) or DFX at concentrations of 10, 50, or 100 μM for 48 h. Immediately after the incubation, caspase 3/7 activity was measured using a luminescence assay and corrected for cell viability determined using the MTS assay. The corrected caspase 3/7 activities of BxPC-3, HPAF-II, and Panc 10.05 cells incubated with DFX increased in a dose-dependent manner. The data are presented as the mean ± SD (*n* = 3). *p <0.05, **p <0.01 vs. control

To further characterize the mechanisms of the antiproliferative activity of DFX, the pancreatic cancer cell lines BxPC-3, HPAF-II, and Panc 10.0 were incubated with either the vehicle control (PBS) or concentrations of 10, 50, or 100 μM of DFX for 72 h, and apoptosis was examined by flow cytometry using PI and Annexin V staining. The results are shown in Fig. 3a. The amount of live cells was defined as the number of cells negative for both Annexin V and PI. The amount of cells in early apoptosis was defined as cells positive for Annexin V only, whereas late apoptosis was defined as cells positive for both Annexin V and PI. The amount of necrotic cells was defined as the cells negative for Annexin V but positive for PI. The percentages of live, apoptotic, and necrotic cells are shown in Fig. 3b. Incubation with 50 or 100 μM DFX significantly decreased the number of live cells compared with control cells in all three cell lines. Moreover, incubation with 50 or 100 μM DFX typically increased the number of cells in late apoptosis in all three cell lines. Apoptosis was also examined by measuring the caspase 3/7 activity with a luminescence assay. The analyzed results are shown in Fig. 4. In all three cell lines, the caspase 3/7 activities were significantly higher in cells incubated with 100 μM of DFX compared with control cells. These results demonstrated that 50 and 100 μM DFX induced apoptosis in pancreatic cancer cells.

### DFX inhibited the growth of human pancreatic cancer xenografts

**Fig. 5:**
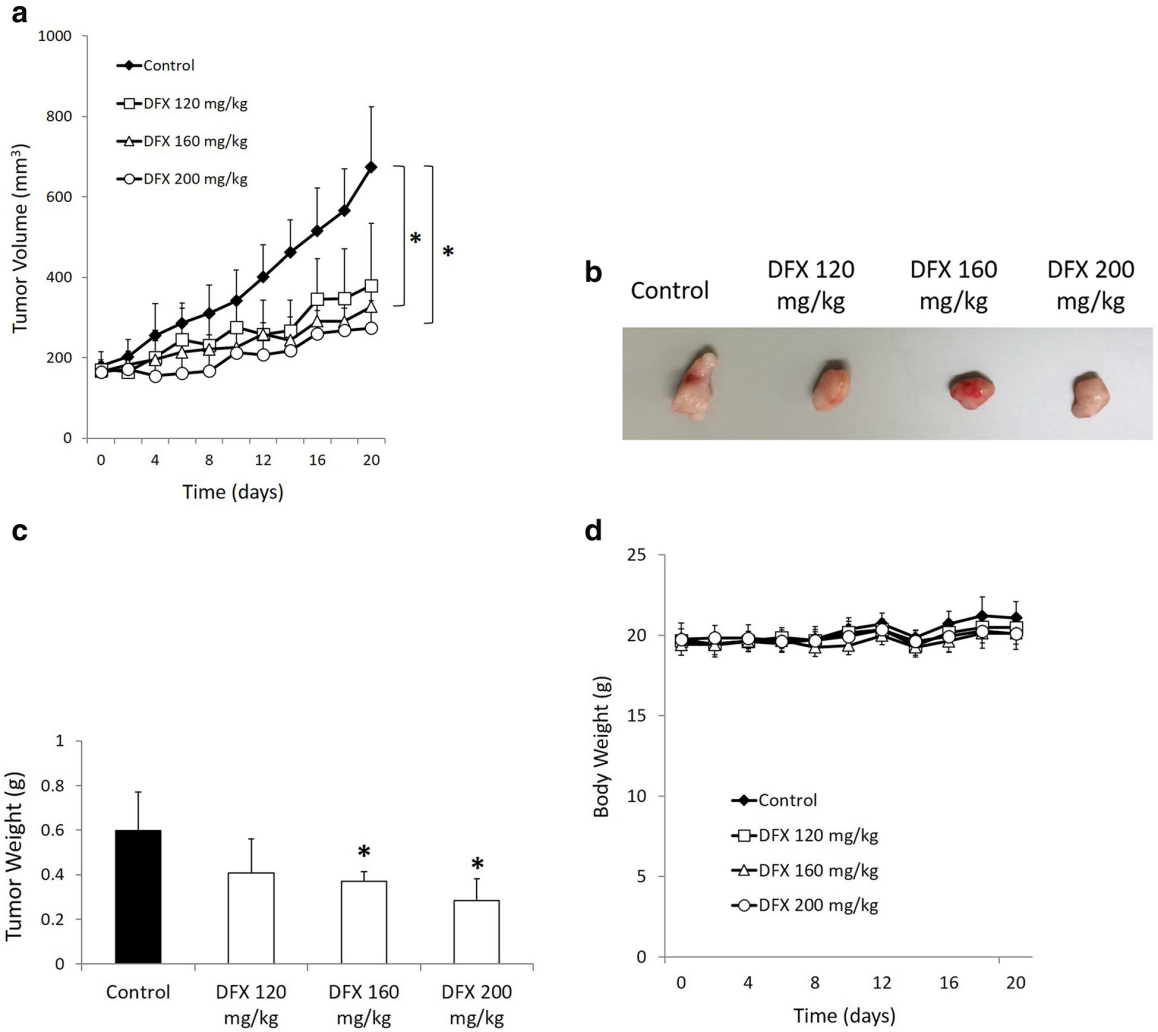
Orally administered DFX markedly inhibited the growth of pancreatic cancer xenografts in nude mice. **a** DFX (160 and 200 mg/kg orally, given by gavage every second day, for a total of three treatments per week for 21 days) significantly inhibited the growth of human pancreatic cancer BxPC-3 xenografts in vivo. **b** The removed tumors were measured and processed for immunohistochemistry and genetic analyses. **c** The removed tumors from mice treated with 160 and 200 mg/kg oral DFX weighed significantly less than the control tumors. **d** The average weight of mice in each treatment group during the course of treatment

**Fig. 6:**
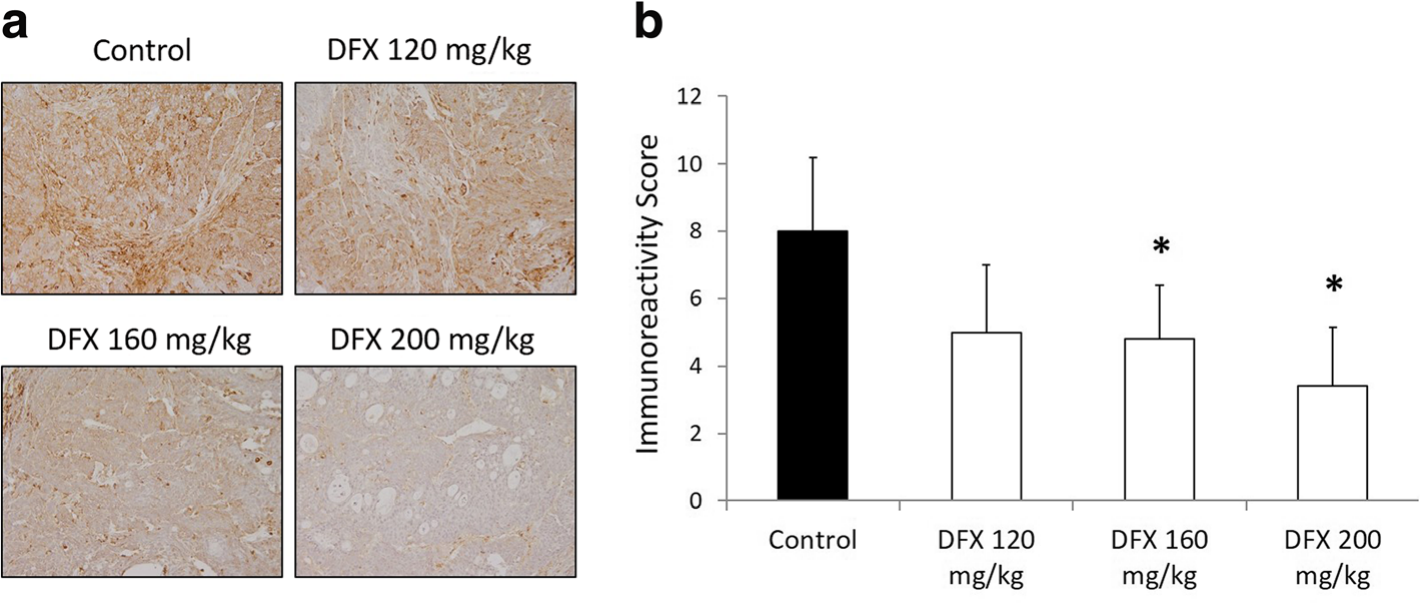
Orally administered DFX reduced ferritin-H protein levels of removed tumors in immunohistochemical analyses. **a** Immunohistochemistry was performed on the removed tumors with antibody specific to ferritin-H. **b** The slides were scored for the percentage of positive cells (0 = 0–5, 1 = 6–25, 2 = 26–50, 3 = 51–75 and 4 = 76–100 %) and intensity (0 = negative, 1 = weak, 2 = moderate, 3 = strong). The immunoreactivity score was calculated as the percentage of positive cells multiplied by the score for the staining intensity. The immunoreactivity scores of removed tumors treated orally with 160 and 200 mg/kg of DFX were significantly lower than that of control tumors. The data are presented as the mean ± SD (*n* = 5 mice per group). For statistical analysis, each treatment was compared with the control. *p <0.05, **p <0.01 vs. control

Next, the antiproliferative activity of DFX against pancreatic cancer was assessed in vivo using BxPC-3 pancreatic cancer xenografts in BALB/c nude mice. As DFX is given to patients orally, we administered DFX as a saline suspension given orally in accordance with previous studies [22, 23]. DFX administered orally at 160 and 200 mg/kg (every second day, three treatments per week for 21 days) resulted in marked inhibition of tumor growth as determined by measurements of tumor volume and tumor weight (Fig. 5a, b, and c). After 21 days of oral treatment with the vehicle control (saline solution), the tumor xenografts reached an average volume of 674 ± 150 mm^3^. In contrast, the tumor volumes were significantly reduced to 327 ± 45 and 274 ± 67 mm^3^ in mice treated with 160 and 200 mg/kg DFX, respectively (Fig. 5a). At the end of the experiment, the tumors were excised and measured. The control tumors weighed 0.6 ± 0.2 g, whereas tumors treated with 160 and 200 mg/kg oral DFX weighed significantly less than the control tumors at 0.4 ± 0.04 and 0.3 ± 0.1 g, respectively (Fig. 5c). Furthermore, in the blood sample examinations, DFX administered orally at 160 and 200 mg/kg for 3 weeks significantly decreased serum levels of ferritin to 8.6 ± 1.5 and 9.8 ± 1.5 ng/ml, respectively, compared with mice that received vehicle alone (18.3 ± 1.9 ng/ml; Table 2). While DFX administered at 160 and 200 mg/kg inhibited tumor growth and decreased the serum levels of ferritin, the mice did not show body weight loss or altered serum biochemistry, with the exception of the serum levels of ferritin (Fig. 5d and Table 2). On the other hand, DFX administered at 120 mg/kg did not significantly inhibit tumor growth, compared with mice administered vehicle alone. Additionally, it is important to note that DFX administered at 120 mg/kg also failed to reduce the serum levels of ferritin in mice. These observations are consistent with immunohistochemical studies on tumor xenografts that performed semi-quantitative analyses of tumor sections. While tumors treated with 160 and 200 mg/kg oral DFX significantly reduced ferritin-H protein levels compared with tumors treated with the vehicle alone, tumors treated with 120 mg/kg oral DFX did not significantly decrease the ferritin-H protein levels compared with tumors treated with the vehicle alone (Fig. 6a and b). These data indicated that tumor growth could be suppressed when tumors were treated with a sufficient dose of DFX, which functions as an iron chelator.

**Table 2:** Serum indices from nude mice bearing a BxPC-3 xenograft that were treated orally by gavage with either the vehicle control or DFX (120, 160, or 200 mg/kg) every second day (three treatments per week) for 21 days

	Units	Treatment groups			
		Vehicle control	Deferasirox		
			120 mg/kg	160 mg/kg	200 mg/kg
Ferritin	ng/ml	18.3 ± 1.9	20.6 ± 2.9	8.6 ± 1.4*	9.8 ± 1.5*
Total protein	g/dl	5.0 ± 0.3	5.4 ± 0.3	5.3 ± 0.4	5.3 ± 0.3
Albumin	g/dl	3.2 ± 0.2	3.3 ± 0.1	3.4 ± 0.1	3.4 ± 0.1
Aspartate aminotransferase	U/l	89.2 ± 15.8	106.2 ± 32.6	155.2 ± 97.9	146 ± 58.9
Alanine transaminase	U/l	23.6 ± 1.9	29.2 ± 8.2	28.4 ± 9.6	28.8 ± 7.2
Lactate dehydrogenase	U/l	243.6 ± 37.9	242.6 ± 18.2	243.8 ± 23.1	243.6 ± 21.7
Blood urea nitrogen	mg/dl	560.6 ± 112.7	578.2 ± 65.5	669 ± 43.9	689.8 ± 101.9
Creatinine	mg/dl	17.6 ± 1.2	14.2 ± 2.1	14.4 ± 0.6	15.8 ± 1.3

*p <0.05 vs. control

### DFX downregulated genes in the pancreatic adenocarcinoma signaling pathway

**Fig. 7:**
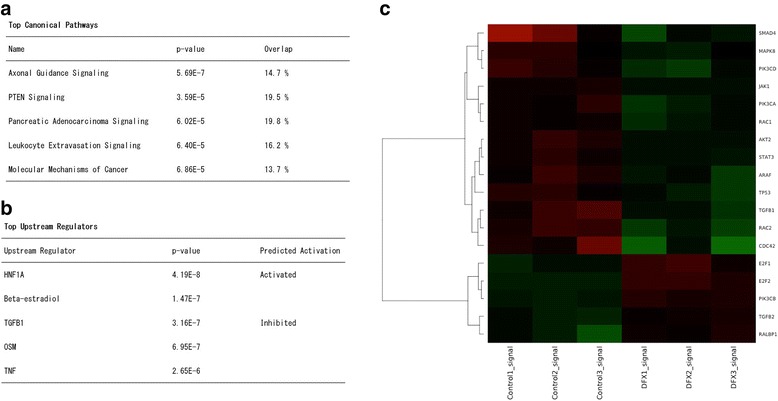
DFX downregulated the genes in pancreatic adenocarcinoma signaling. A total of six tumors, three tumors from mice treated with 200 mg/kg oral DFX, and three tumors from the controls, were chosen to examine gene expression alternation. A total of 2412 genes differentially expressed with a significance cutoff of *p* <0.05 were imported into the IPA. **a** Top canonical pathways by DFX treatment in the removed tumors. Pancreatic adenocarcinoma signaling was observed. **b** Top upstream regulators by DFX treatment in the removed tumors; TGF- ß1 was strongly inhibited. **c** A heatmap of differently expressed genes in the Pancreatic Adenocarcinoma Signaling pathway. Most of the genes were downregulated after DFX treatment

To investigate the genetic effect of DFX in pancreatic cancer, we examined gene expression alternations in the removed tumors exposed to DFX. From the results of the cancer xenograft experiments, we found that the tumors treated with 200 mg/kg oral DFX were suitable for examining gene expression alterations. Thus, three tumors were randomly chosen from the tumors treated with 200 mg/kg oral DFX, and another three tumors were randomly chosen from the control tumors. After the whole genome microarray analysis, a total of 2412 genes were recognized as differentially expressed with a significance cutoff of *p* <0.05. These genes were imported into the IPA, and pathway analyses were performed. The top canonical pathways are shown in Fig. 7a. Pancreatic adenocarcinoma signaling was identified as one of the top canonical pathways. This observation indicated that DFX strongly affected xenografted pancreatic cancer genetically. A heatmap of differently expressed genes included in pancreatic adenocarcinoma signaling is shown in Fig. 7c. Genes highlighted in red indicate upregulation versus the control tumors, while green indicates downregulation in the treated tumors. According to the heatmap, most genes in the pancreatic adenocarcinoma signaling pathway were downregulated by DFX. Specifically, transforming growth factor-ß1 (TGF- ß1) was strongly inhibited. The top upstream regulators are shown in Fig. 7b; TGF- ß1 was also a top upstream regulator. These data demonstrated that the antiproliferative activities of DFX were sustained genetically.

## Discussion

The antiproliferative activity of iron chelators was first demonstrated on leukemia in cell cultures and clinical trials [24, 25]. Then, the antiproliferative activity of iron chelators was demonstrated in solid tumors, including pancreatic cancer tumors, and in cell culture in recent studies [15, 26, 27]. DFO was the first commercially available iron chelator to be used for the treatment of iron-overload disease [28]. DFO has also been used for studies researching the antiproliferative activity of iron chelators in cell cultures and clinical trials [13–15, 25–27]. Although DFO exhibits antiproliferative activity, this chelator has serious limitations because it is not utilized by the body if administered orally and has a short serum half-life. DFO needs to be given parenterally (either subcutaneously or intravenous infusion) for long periods, typically 8–12 h per day, which has led to poor patient compliance. On the other hand, DFX, a recently identified iron chelator, can be administered orally once daily because it is orally active and has a long half-life of 7–18 h. DFX is currently used for the treatment of iron-overload disease and is considered an alternative to DFO [16]. The antiproliferative activity of DFX has been investigated in various cancers [22, 23, 29, 30]. However, there have previously been no studies of the effects of DFX in pancreatic cancer; this study is the first to elucidate the antiproliferative activity of DFX against pancreatic cancer cells.

We examined the in vitro antiproliferative activity of DFX using an MTS assay in three pancreatic cancer cell lines: BxPC-3, HPAF-II, and Panc 10.05. We observed a dose-dependent antiproliferative activity of DFX in pancreatic cancer cell lines, consistent with the results of previous studies in esophageal cancer cell lines [22] or lung cancer cell lines [23]. Although a number of studies have attempted to elucidate the anti-cancer mechanisms of iron chelators, their mechanisms are not well known [12]. Especially in pancreatic cancer, there have been few studies investigating the effect of iron chelators as anticancer agents [15]. To investigate the mechanisms of the antiproliferative activity of DFX, we examined the effects of DFX on the cell cycle and apoptosis in pancreatic cancer cell lines. We observed that 10 μM DFX inhibited pancreatic cancer cell proliferation by arresting the cell cycle in the S phase, and 50 and 100 μM DFX inhibited pancreatic cancer cell proliferation by inducing apoptosis. These anti-cancer mechanisms of DFX are consistent with those found in previous reports for most iron chelators [15, 31, 32].

We next assessed the ability of DFX to inhibit pancreatic cancer growth in vivo using a murine xenograft model. We administered DFX at doses of 120, 160, and 200 mg/kg every second day, totaling three treatments per week for 3 weeks. The doses of 160 and 200 mg/kg of DFX successfully inhibited tumor growth and decreased serum and tumor levels of ferritin. Initially, we attempted to administer DFX at doses of 20–40 mg/kg every second day, for three treatments per week for 3 weeks because a 20 mg/kg per day regimen is considered suitable in patients with iron overload [33]. However, in nude mice, 20–40 mg/kg DFX did not inhibit tumor growth or reduce serum levels of ferritin (data not shown). In fact, even a dose of 120 mg/kg of DFX failed to significantly suppress either tumor growth or serum and tumor ferritin levels. The 3-week experiment may have been too short to assess the effects of a normal dose of DFX in this xenograft model. However, it is important to note that decreased serum and tumor levels of ferritin were observed in the mice that received 160 or 200 mg/kg doses of DFX administration, and the xenografted tumors were markedly suppressed. Furthermore, no serious effects on body weight and biological indices were observed. A previous in vivo study using DFX also demonstrated the importance of iron depletion in the xenografted tumor for cancer therapy [22]. According to our study, we believe that DFX demonstrates antiproliferative activity by decreasing serum levels of ferritin, which is reflected as iron depletion in the tumor.

To assess the genetic effects of DFX for pancreatic cancer, we conducted microarray analysis using in vivo samples. Most genes included in pancreatic adenocarcinoma signaling, especially TBF- ß1, were downregulated by DFX administration. A previous study revealed that TGF- ß overexpression is associated with early recurrence following resection and decreased survival in patients with pancreatic cancer [34]. TGF- ß1 also plays pivotal roles in driving epithelial-mesenchymal transition (EMT) in the pathogenesis of pancreatic cancer [35, 36]. In fact, the TGF- ß signaling inhibitor displays antiproliferative activity for pancreatic cancer [37]. A recent review article also demonstrated that iron chelators can target several pathways, including the TBF- ß pathway, to subsequently inhibit cellular proliferation, EMT and metastasis [38]. This evidence, combined with the results of our microarray analysis, indicates that DFX works as anticancer agent by suppressing TGF- ß signaling.

## Conclusions

We first elucidated that DFX has potential as a therapeutic agent for pancreatic cancer. We demonstrated that DFX inhibits pancreatic cancer cell growth by arresting the cell cycle and inducing apoptosis. Furthermore, DFX inhibited pancreatic cancer growth in vivo in a murine xenograft model. Genetically, TGF- ß1 plays a key role in the effect of DFX against pancreatic cancer. Because DFX is a commercially available oral iron chelator, its clinical application can be considerable. While further extensive studies are required, the DFX treatment strategy can be considered a novel effective and safe pancreatic cancer therapy in the near future.

## Abbreviations

DFO: Deferoxamine
DFX: Deferasirox
EMT: Epithelial-mesenchymal transition
IPA: Ingenuity pathway analysis
MTS: 3-(4,5-dimethylthiazol-2-yl)-5-(3-carboxymethoxyphenyl)-2-(4-sulfophenyl)-2H-tetrazolium, inner salt
PBS: Phosphate-buffered saline
PI: Propidium iodide
TGF- ß: Transforming growth factor-ß
